# Preparation of Mesoporous Silica Nanosphere-Doped Color-Sensitive Materials and Application in Monitoring the TVB-N of Oysters

**DOI:** 10.3390/foods11060817

**Published:** 2022-03-12

**Authors:** Binbin Guan, Fuyun Wang, Hao Jiang, Mi Zhou, Hao Lin

**Affiliations:** 1 Nantong Food and Drug Supervision and Inspection Center, Nantong 226400, China; guanbinbinde@126.com (B.G.); ntsyj_zm@126.com (M.Z.); 2 School of Food and Biological Engineering, Jiangsu University, Zhenjiang 212013, China; wfy1816491734@163.com (F.W.); jh113997@163.com (H.J.)

**Keywords:** oysters, total volatile basic nitrogen detection, mesoporous silica nanospheres, colorimetric sensor array, pattern recognition

## Abstract

In this work, a new colorimetric sensor based on mesoporous silica nanosphere-modified color-sensitive materials was established for application in monitoring the total volatile basic nitrogen (TVB-N) of oysters. Firstly, mesoporous silica nanospheres (MSNs) were synthesized based on the improved Stober method, then the color-sensitive materials were doped with MSNs. The “before image” and the “after image” of the colorimetric senor array, which was composed of nanocolorimetric-sensitive materials by a charge-coupled device (CCD) camera were then collected. The different values of the before and after image were analyzed by principal component analysis (PCA). Moreover, the error back-propagation artificial neural network (BP-ANN) was used to quantitatively predict the TVB-N values of the oysters. The correlation coefficient of the colorimetric sensor array after being doped with MSNs was greatly improved; the Rc and Rp of BP-ANN were 0.9971 and 0.9628, respectively when the principal components (PCs) were 10. Finally, a paired sample *t*-test was used to verify the accuracy and applicability of the BP-ANN model. The result shows that the colorimetric-sensitive materials doped with MSNs could improve the sensitivity of the colorimetric sensor array, and this research provides a fast and accurate method to detect the TVB-N values in oysters.

## 1. Introduction

Oysters are marine organisms known as “ocean milk”, which represent an important part of global seafood consumption. They are rich in protein and trace elements which are necessary for human body. They are deeply loved by consumers and the global demand for oysters is increasing year by year [1,2]. Unfortunately, oysters are prone to spoilage due to the high contents of water, fat, protein, etc., which are easily used by bacteria and fungi, and deteriorated oysters affect eating quality and food safety [3]. Therefore, it is critical to optimize quantitative measurements of spoilage during transport, storage, and processing to ensure quality. There are many quality characteristics, but the main ones include texture, color, softness, pH, and freshness [4,5]. In particular, freshness is the most important reference index for evaluating quality and safety [6,7,8]. Traditional techniques such as sensory, chemical, and microbial population-assessment techniques are the primary methods used to determine the shelf life or freshness of oysters [9,10]. As human sensory evaluations are susceptible to subjective factors, even for experienced and trained personnel, it is difficult for humans to perform quantitative sensory assessments and to make assessments consistent across raters. Therefore, it is a flawed method in industrial application. With regard to other methods, including chemical techniques (such as gas chromatography–mass spectrometry (GC-MS)) and microbiological measurements, the above detection methods all required pretreatment of samples, which were destructive, cumbersome pretreatment steps, and microbial cultures would inevitably need a lot of detection time, which was time-consuming. Hence, it is meaningful to develop rapid and accurate analytical techniques to assess the freshness of oysters.

During the storage process, the volatile organic compound (VOC) changes in oysters are closely related to freshness. For instance, with the decrease in freshness of oysters, volatile organic compounds (VOCs) such as 2,6-nonadiena, lilac aldehyde, and pentanal are significantly increased [1]. Moreover, VOCs also affect the quality to a certain extent.

GC-MS and sensor technology are the two major detection methods currently used for volatile-gas analysis [11]. GC-MS usually requires sample pretreatments, so the cumbersome operation steps lead to less application in the field of rapid detection [12]. However, sensor technology could obtain the digital outputs by chemical or physical sensors in minutes. Currently electronic-nose technology is the most widely used sensor, and is commonly used in the field of rapid detection [13]. The electronic nose is mainly composed of gas sensor array, signal preprocessing, and pattern recognition. The gas sensor converts the chemical input of a certain gas into an electrical signal which would be processed using suitable pattern-recognition analysis methods after appropriate preprocessing. It has the advantages of a short response time and fast detection speed. However, this method is mainly based on weak physical adsorption, which results in low selectivity and low sensitivity. Moreover, the electronic nose is easily to be affected by humidity, resulting in signal drift [14,15]. The colorimetric sensor array, invented by Kenneth Suslick, is a novel electronic nose which consists of chemical dyes that are sensitive to specific VOCs. Quantitative or qualitative analysis of the VOCs would be detected by the unique color changes in the colorimetric sensor array after reaction with VOCs [16,17]. Colorimetric sensor technology has certain potential and application prospects in the detection of food freshness and harmful substances, and it has been widely used in the evaluation of food quality such as the freshness of meat, alcohol content monitoring of vinegar substrate during the production of vinegar, rice quality, and so on [18,19,20,21].

However, current sensors are mostly unmodified raw commercial color-sensitive materials, and these sensors are difficult to be used for the detection and analysis of one or more specific gases of food that reflect its quality characteristics. Therefore, due to the complexity of VOCs during oysters’ storage, it is difficult to detect the TVB-N by unmodified color-sensitive materials quantitatively. Therefore, it is particularly important to improve the binding force between the sensor substrate and the VOCs to obtain a high-sensitivity and strong-stability colorimetric sensor array during the preparation of the sensor. At present, nanotechnology has been widely used to add special properties to materials [22]. When color-sensitive materials are polymerized with polymer nanospheres, due to the specific surface area effect, small-size effect and interface effect of nanospheres [23,24], nanocolorimetric-sensitive materials with higher sensitivity and chemical activity could be obtained.

In this study, mesoporous silica nanospheres (MSNs) were synthesized and the Scanning Electron Microscope (SEM), Energy Dispersive Spectrometer (EDS), Transmission Electron Microscope (TEM), and BET were used to characterize the morphology of MSNs. Next, the MSNs were used to modify the color-sensitive materials, and the colorimetric sensor arrays doped with MSNs were used to detect the freshness of oysters. Additionally, the total volatile basic-nitrogen (TVB-N) of the oyster samples was determined by the traditional chemical-detection method, and the correlation coefficient between the traditional chemical-detection method and colorimetric sensor array was established by back-propagation artificial neural network (BP-ANN) model. At the same time, the result of the correlation coefficient of the colorimetric sensor array after doped with MSNs was compared with the unmodified colorimetric sensor array. Finally, a paired sample *t*-test was used to verify the difference between the predicted values of the model and measured values.

## 2. Materials and Methods

### 2.1. Materials

Frozen oyster meat was purchased from Zhenjiang Metro Supermarket; all oyster meat samples were relatively uniform in size, weighing 15–18 g/piece. Afterwards, they were placed in a refrigerator at 4 °C for 1 day (group 1), 3 days (group 2), 5 days (group 3), 7 days (group 4), and 9 days (group 5), which were divided into five groups for a total of 150 samples (30 samples of each group), and the samples were not subjected to moisture-absorption treatment. Ethanol, Cetyltriethylammnonium bromide (CTAB), Tetraethoxysilane (TEOS), Polyethylene glycol-600 (PEG-600), N,N-Dimethylformamide (DMF), magnesium oxide (MgO), methyl red, bromocresol green, and 0.1 mol/L hydrochloric acid standard titrant were purchased from J&K Scientific Co., Ltd. (Shanghai, China). Deionized water was obtained by laboratory ultrapure water machine (Milli-Q Direct 8, Merck Millipore, Darmstadt, Germany).

### 2.2. Synthesis of MSNs

Based on the improved Stober method, mesoporous silica nanospheres (MSNs) were synthesized [25]. 100 mL absolute ethanol was mixed with 300 mL deionized water in a 500 mL three-necked flask, and then 6 mL of ammonia was added to the above mixture. The whole system was continuously stirred at 25 °C, and 25 mg of CTAB was quickly added. Then, 0.2 g TEOS was added after the solution had been uniformly mixed. The reaction condition was maintained for 12 h to ensure a complete reaction. Finally, the suspension obtained by filtration was calcined in a muffle furnace at 540 °C for 6 h to remove the template, and white powders were obtained after calcination, which were MSNs. Scanning Electron Microscope (SEM) (ZEISS Gemini SEM 300, Carl Zeiss AG, Jena, Germany), Energy Dispersive Spectrometer (EDS) (X-MaxN50, Oxford, UK), Transmission Electron Microscope (TEM) (JEM-2100F, JEOL, Tokyo, Japan) and BET (Asap 2460, Micromeritics, Norcross, GA, USA) were used to characterize the morphology of MSNs.

### 2.3. Color-Sensitive Materials Doped with MSNs

The color-sensitive materials (CSMs) used in this study were synthesized in this laboratory, which referred to the classic Lindsey methodology [21,26]. Based on previous experiment, nine CSMs including ZnTPP, MnTPP, Doil, pCarBDP, NO_2_Br_2_BDP, NaiOCH_3_BDP, (HBDP)_2_Ni(II), (C_2_H_4_O_2_BDP)_2_Ni(II), (NaiOCH_3_BDP)_2_Ni(II), which were sensitive to the freshness characteristic VOCs of oysters were screened out and doped with mesoporous silica nanospheres.

**Figure 1:**
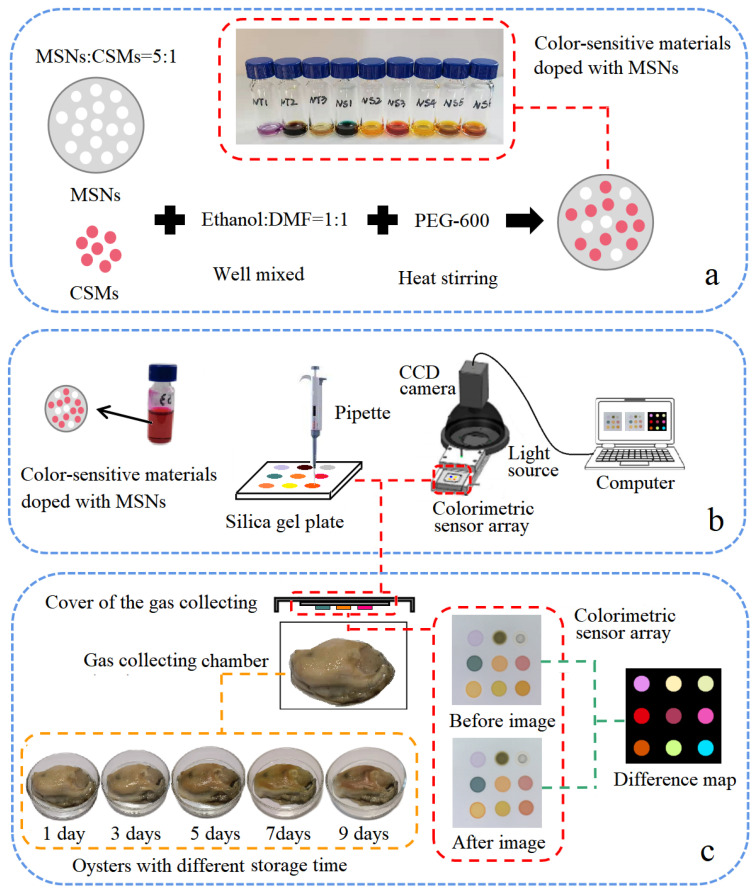
The assembly process of CSMs doped with MSNs (**a**), the schematic diagram of colorimetric sensor array system (**b**) and the difference map acquisition of oysters (**c**).

As shown in Figure 1a, MSN-mixed CSMs (the mass ratio was 5:1, and the masses of MSNs and GMS were 10 mg and 2 mg, respectively) were dispersed in 800 μL DMF and ethanol (volume ratio is 1:1, and the volumes of DMF and ethanol were both 0.4 mL), then 200 μL PEG-600 and a stirrer were added; the mixture was stirred at 350 r/min for 10 min at 50 °C, then the temperature was raised to 90 °C at a rate of 5 °C/5 min and maintained for 60 min. After the reaction was completed, the assembled color-sensitive material was cooled to room temperature for use.

### 2.4. Colorimetric Sensor Array Data Acquisition

As shown in Figure 1b, 9 kinds of color-sensitive materials doped with MSNs were printed on a 3 cm × 3 cm silica gel plate (Merck, Darmstadt, Germany) by a pipette (2.5 μL), constructing a 3 × 3 colorimetric sensor array. The schematic diagram of colorimetric sensor array is shown as Figure 1b; the device used a spherical light source to ensure the uniform image brightness, the CCD camera acquired the image of the colorimetric sensor array before it exposed to the VOCs of the oysters, and it was recorded as the "before image", as seen from Figure 1c, after the "before image" was recorded. After the colorimetric sensor array was fully contacted with the VOCs of oysters for 10 min, the CCD camera recorded the image as “after image”. In this study, 150 oyster samples were divided into 5 groups with different storage times (30 samples stored at 4 °C for 1 day, 3, 5, 7, and 9 days, respectively). After the reaction, the specific image-processing software processed the image to obtain the average gray value of R, G, and B components before and after the reaction of the color-sensitive material in the region of interest (ROI) area, as well as the difference between the reaction values. Among the 150 oyster samples, a random three-fifths (90 samples) were used as training-set samples and the remaining two-fifths (60 samples) were used as prediction-set samples. In addition, the R, G, and B components of each color-sensitive material doped with MSNs (a total of 27 variables) were used in statistical and subsequent pattern recognition.

### 2.5. TVB-N Analysis

The total volatile basic-nitrogen (TVB-N) of the 150 samples was determined by automatic Kjeldahl method, which can refer to Chinese standard GB 5009.228-2016 [27]. After characterization of the VOCs by colorimetric sensor array, the samples were crushed. A total of 10 g of the edible parts of oysters was taken into a distillation tube, then blended with 75 mL distilled water and shaken well and impregnated still for 30 min. Then, 1 g of magnesium oxide (MgO) was added to the distillation tube containing the processed sample, and was immediately connected to the distiller; the receiving solution with a concentration of 20 g/L was 30 mL, which was added with 10 drops of mixed-indicator solution (methyl red ethanol solution:bromocresol green ethanol solution = 1:5), the distillation time was set as 180 s, and finally the receiving solution was titrated with 0.1 mol/L hydrochloric acid standard titrant. TVB-N content was calculated and expressed with a unit of mg/100 g.

### 2.6. Multivariate Statistical Analysis

Multivariate analysis methods played a key role in characterizing TVB-N in oyster samples based on the colorimetric sensor array. The PCA was implemented in Matlab R2016b, the BP-ANN algorithm was implemented in NeuroShell 2, and the paired sample *t*-test was implemented in SPSS Statistics 17 (SPSS, Chicago, IL, USA).

## 3. Results

### 3.1. Variation Trend of TVB-N during Oysters’ Storage

Due to the action of enzymes and bacteria in the process of spoilage in animal foods, the proteins in animal foods are broken down into amines and other volatile alkaline nitrogenous substances, called total volatile base-nitrogen (TVB-N).

**Figure 2:**
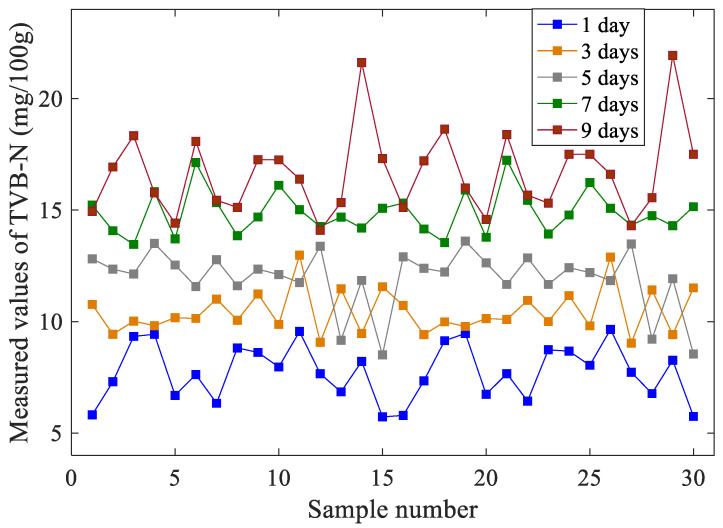
The TVB-N values of oysters with different storage times.

As shown in Figure 2, as the storage time increased, the TVB-N values of oysters also increased, which was proportional to the freshness of oysters. However, there was a certain overlap between the TVB-N values of oyster samples with different storage days. This is because the individual oyster samples are independent and there are certain differences between them.

### 3.2. Characterization of MSNs

**Figure 3:**
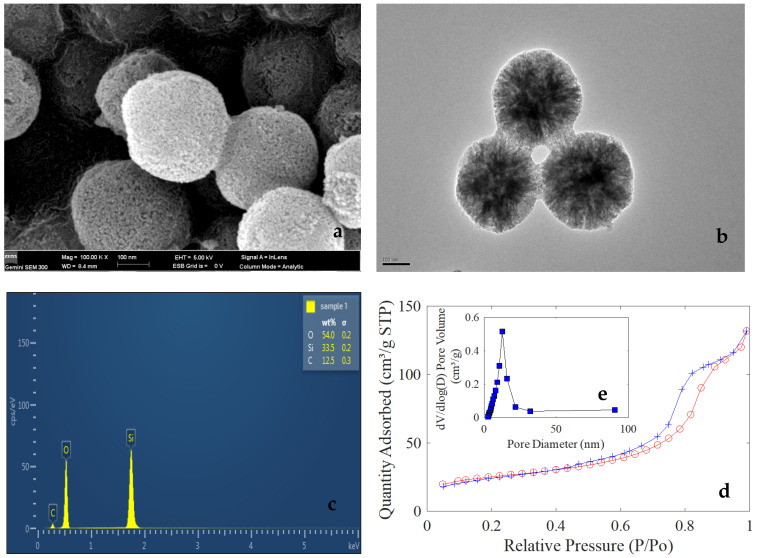
The SEM image of MSNs (**a**), TEM image of MSNs (**b**), EDS image of MSNs (**c**), adsorption–desorption hysteresis lines of MSNs (**d**), and the pore size distribution of MSNs (**e**).

Typical SEM and TEM images of the white powder were shown in Figure 3. As can be seen from Figure 3a,b, the white powder after calcination is composed of spherical nanoparticles with a diameter of about 250 nm, and these nanoparticles have porous characteristics. The corresponding EDS characterization result is shown in Figure 3c, which indicates that the presence of Si and O elements and the spherical nanoparticles may be composed of silica, and the few presences of C element in Figure 3c is due to the presence of hydrocarbons in the air as well as the large amount of carbon in the conductive tape.

The BET result shows that the surface area of the white powder is 84.1618 m^2^/g. Figure 3d shows typical H1 adsorption-desorption hysteresis lines, and indicates the presence of a system in which the porous adsorbent exhibits capillary condensation. In addition, there is a saturated adsorption plateau on the adsorption isotherm of the H1-type hysteresis loop, reflecting that the pore size distribution is relatively uniform, which is generally easy to be observed in the spherical mesoporous material aggregated with relatively uniform size [28]. Figure 3e shows the pore size distribution of white nanospheres; the pore sizes of PSNs are concentrated around 12 nm. All the above characterization results indicate that the MSNs have an adsorption capacity which could accommodate small molecules for chemical reactions.

### 3.3. Image Characterization of Oysters Stored for Different Times by Colorimetric Sensor Array

**Figure 4:**
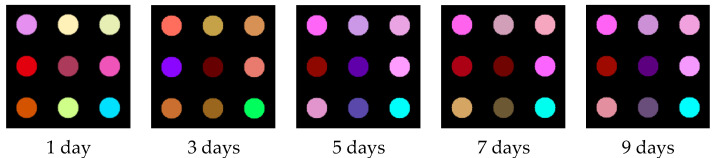
Difference maps of colorimetric sensor arrays after the reaction of the oysters during the storage.

Average color-change profiles were obtained from oyster samples with different storage times. Figure 4 shows the difference maps of the VOCs of oyster samples with a storage time of 1, 3, 5, 7, and 9 days exposed to colorimetric sensor array doped with MSNs. As shown in Figure 4, the different maps of colorimetric sensor arrays with different storage-time oysters present different color difference maps (The difference map of the sensor array was obtained after superimposing the gray image of R, G, and B components: for example, changes in the B-component average values for the ninth nanocolorimetric-sensitive material-oyster samples stored for different days were 4.93, 24.02, 20.40, 26.07, and 23.03, respectively), and each dye has its specific fingerprint after the reaction with different samples. The difference map is an overall characterization of the overall VOCs of oysters. Since the VOCs of the oysters at 1, 3, and 5 days are constantly changing, the difference maps of these three days are constantly changing. With the increase in storage days, oysters were spoiled and there was a lot of TVB-N, for which, as the main component of the VOCs, the color difference maps show some similarity in 5, 7, and 9 days.

### 3.4. PCA Analysis

There were 27 variables in this study; this excessive number of variables would increase the complexity of statistical analysis. Furthermore, the 27 variables of the colorimetric sensor contained a lot of overlapping information. Principal component analysis (PCA) is a multivariate statistical analysis method, which could select a few important variables from multiple variables by linear transformation, using orthogonal transformation transform a set of potentially correlated variables into a set of linearly uncorrelated variables [28]. It is more similar to a preprocessing method, which can simplify data well. This group of variables after conversion are called ‘principal components’. They take the data points (changes in RGB values for each nanocolorimetric-sensitive material) from all the oyster samples and generate a set of orthogonal eigenvectors (principal components, PCs) for maximum variance.

**Figure 5:**
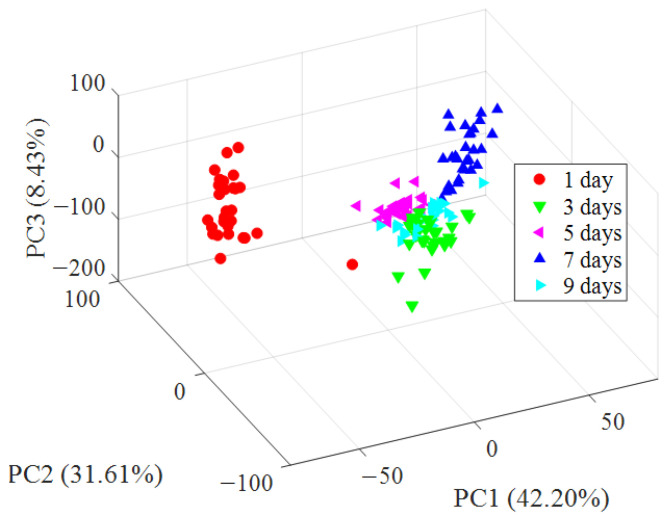
Classification result achieved by PCA.

A 3D space of all oyster samples are shown in Figure 5, which shows the scatter plots of oysters which are represented by PC1 (42.20%), PC2 (31.61%), and PC3 (8.43%); the first three principal components reached 82.24%, which represents most variables’ information. As can be seen from Figure 5, the oyster samples of different storage days have a certain clustering trend in the figure, especially the oysters that had been stored for 1 day, which can be completely distinguished from other oysters in the PCA score-plots figure. Although the oyster samples in the late storage period have a certain intersection in the clustering, they still show a certain clustering trend. These phenomena can be explained, as on the one hand, the oyster samples have certain differences themselves, and on the other hand, corruption is a continuous process.

### 3.5. Quantitative Analysis of Colorimetric Sensor Array for TVB-N Detection in Oysters

The back-propagation artificial neural network (BP-ANN) model is established with the principal components (PCs) after PCA analysis as the model input and the TVB-N values measured by automatic Kjeldahl method as the network output [29] with a random sample of three-fifths of all the 150 samples (90 samples) as the calibration set and the remaining 60 samples for the prediction-set data. The number of training iterations is set to 100; the transfer function from the input layer to the hidden layer and from the hidden layer to the output layer is the tanh function; the network learning rate is 0.1; the momentum factor is 0.1; and the initial weight value is 0.3.

**Figure 6:**
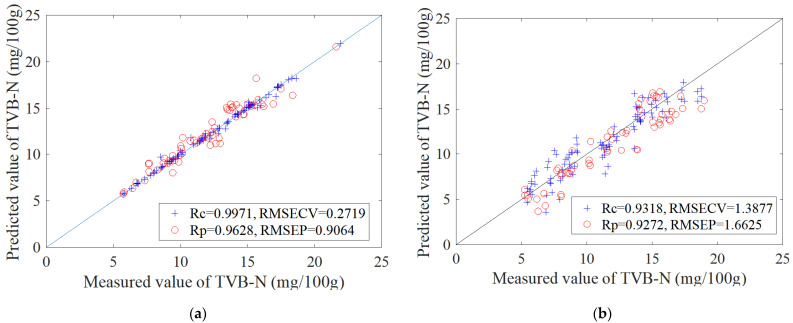
TVB-N correlation between Kjeldahl nitrogen-measured values and the prediction value by color-sensitive materials doped with MSNs (**a**); and color-sensitive materials without MSN doping (**b**).

Table 1 shows the BP-ANN results of TVB-N concentration under 6–12 PCs. It can be seen from Table 1 that when the PCs are 10, the correlation coefficient of TVB-N in the prediction set (Rp) was the highest; at this time, the correlation coefficient in the calibration set (Rc) was 0.9971. Figure 4 shows the scatter plot showing a correlation between Kjeldahl nitrogen-measured values and colorimetric sensor array prediction values combined with the BP-ANN model. The signs of blue plus and red circles respectively represent the calibration and prediction data. As seen from Figure 6a, the results of measured TVB-N values and colorimetric sensor array prediction values are highly correlated in BP-ANN model after being doped with MSNs. The root mean square error of the cross-validation (RMSECV) value was 0.2719 while Rc was 0.9971; the root mean square error of the prediction (RMSEP) value was 0.9064 while Rp was 0.9628. Figure 6b shows the correlation between the measured values and the prediction values by color-sensitive materials without MSN doping. The RMSECV value was 1.3877 while Rc was 0.9318; the RMSEP value was 1.6625 while Rp was 0.9272. Compared with the results of the color-sensitive materials without MSN doping, the correlation coefficients of the calibration set and the prediction set were both significantly improved after doping, while the root mean square error of the calibration set and the prediction set was significantly reduced. Therefore, after doping with MSNs, the VOC messages of oysters with different storage times collected by colorimetric sensor array present a high relevance with TVB-N values measured by the Kjeldahl nitrogen method.

### 3.6. The Accuracy Test of Formaldehyde Quantitative Model Paired-Sample t-Test

To verify the accuracy and applicability of the BP-ANN model, a paired-sample *t*-test was used to verify the pairwise difference between the predicted value of TVB-N in the BP-ANN model and the measured value of automatic Kjeldahl nitrogen determination. As can be seen from Table 2, Sig. value of the training set and prediction set were both bigger than 0.05; hence, there was no significant difference between the model’s predicted values and measured values. As a result, it can be considered as a rapid, simple, visualization-detection method for TVB-N value detection of oysters with different storage times.

## 4. Conclusions

This paper presents a novel method for TVB-N value detection in oysters which is based on color-sensitive material doped with MSNs. Response data of the before and after image were first analyzed with PCA analysis; the results indicate that the different freshness of oyster samples shows different clustering trends in the PCA score plots figure, especially fresh oysters, which could be directly and completely distinguished from the rest of the oysters in the picture. Then, BP-ANN was used to quantitatively predict the TVB-N values of oysters during the storage process; the result shows that when PCs were 10, the correlation coefficient in the prediction set (Rp) was the highest and the Rc and Rp were 0.9971 and 0.9628, respectively. In addition, compared with the color-sensitive material without nanoassembly, the correlation coefficient of the colorimetric sensor array after doping with MSNs was greatly improved; this may be due to the specific surface-area effect and the interface effect of the mesoporous silica nanospheres, and after doping with MSNs, the nano-colorimetric sensitive materials obtained a higher chemical activity. Finally, a paired-sample *t*-test was used to verify the accuracy and applicability of the BP-ANN model; the result showed that there was no significant difference between the results of the BP-ANN model and automatic Kjeldahl nitrogen determination. Therefore, this research provides an objective, accurate, environmentally friendly, and quantifiable method to detect TVB-N values in oysters. In addition, it provides a method to improve the sensitivity of the colorimetric sensor array. Furthermore, the mechanism analysis of colorimetric-sensitive materials after binding with trimethylamine based on quantum-chemistry calculation is under study.

## Figures and Tables

**Table 1:** BP-ANN model results of TVB-N with different PCs.

PCs	Rc	Rp
6	0.9920	0.8932
7	0.9857	0.9076
8	0.9807	0.9156
9	0.9999	0.9420
10	0.9971	0.9628
11	1.0000	0.9504
12	0.9945	0.9526

**Table 2:** The results of paired-sample *t*-test.

Samples		Training Set	Prediction Set
Pairwise difference	Mean	−0.00114	−0.13443
	Standard deviation	0.27342	0.90393
	Standard error of the mean	0.02882	0.11670
	t	−0.039	−1.152
	df	89	59
	Sig.	0.969	0.254

## Data Availability

Not applicable.
